# Electronic structures and optical properties for Ag-N-codoped ZnO nanotubes

**DOI:** 10.1186/1556-276X-8-365

**Published:** 2013-08-27

**Authors:** Xian-Yang Feng, Chang-Wen Zhang, Xi-Jin Xu, Pei-Ji Wang

**Affiliations:** 1 School of Physics and Technology, University of Jinan, Jinan 250022, People's Republic of China

**Keywords:** Ag-N codoped, ZnO nanotube, Electronic structure, Optical property

## Abstract

The structural and electronic/optical properties of pure and Ag-N-codoped (8,0) ZnO nanotubes have been studied using first-principles calculations in the framework of the local spin density approximation. The configurations for Zn atoms replaced by Ag atoms are p-type semiconductor materials, and the bandgap increases when N atoms are doped into ZnO nanotube configurations. The optical studies based on dielectric function and reflectivity indicate that new transition peaks in the visible light range are observed, which can be ascribed to the Ag and N doping. Furthermore, there is a red shift observed with the increase of N concentration.

## Background

Since the discovery of single-walled carbon nanotubes (SWCNTs) in the early 1990s [1], the research on tubular nanostructures has attracted increasing interest because their unique structures can provide some unique properties, such as high Young's modulus, high thermal conductivity, and high aspect ratio structure. Besides SWCNTs, many other tubular nanostructures such as boron nitride nanotubes, gallium nitride (GaN) nanotubes, and zinc oxide (ZnO) nanotubes have been intensively investigated in recent years. Density functional theory (DFT) calculations have shown that the single-walled GaN, AlN, and InN nanotubes are all metastable, and they are semiconductors with either a direct bandgap (zigzag tubes) or an indirect bandgap (armchair tubes) [2-5].

Recently, Shen et al. found that ZnO single-walled nanotube (SWNT) is more/less stable than its nanowire or nanobelt if the diameter is smaller/bigger than that of (24,0) ZnO SWNT [6]. Hence, the small-diameter (8,0) ZnO SWNT is expected to be more stable. Additionally, Zhou et al. also studied the size- and surface-dependent stability of (8,0) ZnO nanotube, and found that the (8,0) ZnO nanotube had a good surface texture [7].

To get p-type doped ZnO, group V, group IA, and group IB elements have been used as dopants [8-13]. Different doping elements are favorable in O-poor/rich conditions to realize p-type doped ZnO, and the doping will easily produce oxygen vacancy defects. For example, N doping is only favorable in O-poor conditions but will easily produce oxygen vacancy defects. For element Ag, it has smaller diameter and larger ionization energy than group IA elements, and its doping process is favorable in O-rich conditions, which can suppress the defects in ZnO; thus, element Ag is a better candidate for p-type ZnO doping.

Codoping ZnO with transition metal/nonmetal ions is an effective way to modify its electronic/optical properties [14,15]. In this paper, the structure and formation energies of Ag-N-codoped ZnO nanotubes were firstly calculated using DFT and followed by the calculations on the electronic and optical properties with the optimized structures.

## Methods

**Figure 1:**
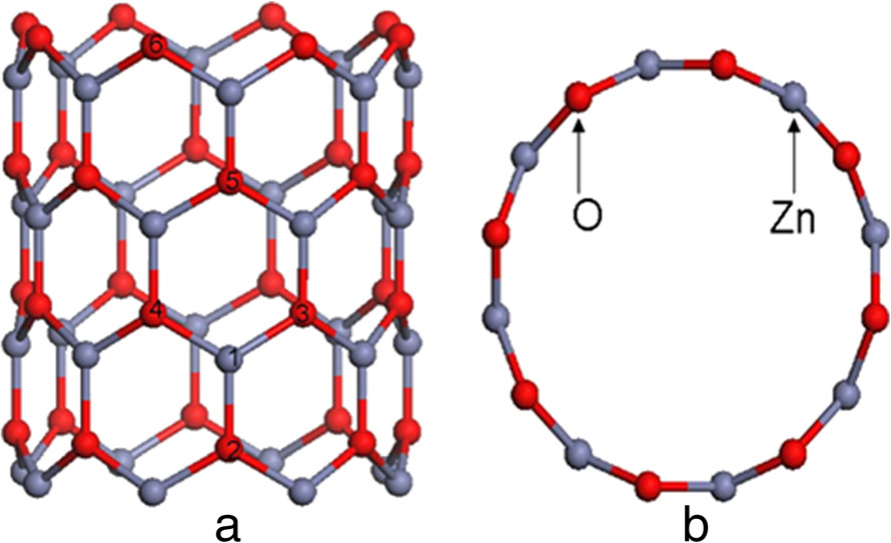
**(8,0) ZnO nanotube. (a)** Ag atom doped at 1 site and N atoms which can be doped at 2, 3, 4, 5, and 6 sites. **(b)** Top view of (8,0) ZnO nanotube. Red and gray balls represent O and Zn atoms, respectively.

Multiwalled and single-walled ZnO nanotubes with similar structures to CNTs can be successfully realized by cutting the atoms inside and outside of ZnO crystalline supercell along the *c* direction. Single-walled ZnO nanotubes can be regarded as the thinnest walled ZnO nanotubes whose structures are similar to CNTs. In our case, the zigzag (8,0) ZnO nanotube containing 64 atoms is selected as a prototype, as shown in Figure 1. Six other configurations based on this structure are considered for the study of the properties of Ag-N-codoped ZnO nanotubes. The first model is obtained by replacing one Zn atom with an Ag atom (Ag atom at 1 site, named as Ag_1_). For the configurations with one and two N atoms replacing two O atoms, the N atoms can be at 2 and 3, 4 sites, which are named as Ag_1_N_2_ and Ag_1_N_3,4_, respectively. The Ag_1_N_5_ and Ag_1_N_6_ configurations are the ones with Ag replacing Zn at 1 site and N replacing O at 5 and 6 sites.

The first-principles full-potential linearized augmented plane wave method based on the generalized gradient approximation [16] is used for the exchange-correlation potential within the framework of DFT to perform the computations, as implemented in the WIEN2K simulation package. Special *k* points were generated with the 1 × 1 × 4 grid based on Monkhorst-Pack scheme. Good convergence was obtained with these parameters. The total energy was converged to be 1.0 × 10^−4^ eV/atom in the optimized structure.

## Results and discussion

### Geometry structures and formation energies

Figure 1 shows the top-view and side-view models of the optimized structures for zigzag single-walled (8,0) ZnO nanotubes. The single-walled ZnO nanotubes are obtained by folding a single-layered graphitic sheet from the polar (0001) sheet of wurtzite bulk structure. Another study showed that the ZnO nanotubes are more stable than ZnO nanowires for small diameters (the number of atoms is smaller than 38 for one unit cell) [6]. Hence, the (8,0) ZnO nanotubes (with 32 atoms in one unit cell) constructed in this paper are reasonable. The formation energies of Ag-N-codoped (8,0) ZnO SWNT were calculated to evaluate their stability. The formation energy can be expressed as

$$E_{f}=E\left(\mathrm{Ag},N-\mathrm{ZnO}\right)-E\left(\mathrm{ZnO}\right)+µ\left(\mathrm{Zn}\right)/µ\left(O\right)-µ\left(\mathrm{Ag}\right)/µ\left(N\right)$$

In this equation, *E*(Ag,N-ZnO) and *E*(ZnO) are the total energies of ZnO SWNTs with and without the impurity, respectively, and *μ* is the chemical potentials of Zn, O, Ag, and N, which depend on the growth conditions. The formation energies are listed in Table 1. The formation energy of Ag-doped ZnO nanotubes is apparently smaller than Ag-doped ZnO nanowires [17], which indicates that Ag-doped nanotubes is more easily achieved than nanowires. For the configurations with N atoms replacing O atoms, the formation energy increases with the increase of N concentration, indicating that low N concentration is more stable. For the configuration with the same N concentration, the Ag_1_N_2_ configuration is more stable than Ag_1_N_5_ and Ag_1_N_6_ configurations. The formation energies of Ag_1_N_2_, Ag_1_N_5_, and Ag_1_N_6_ are smaller than Ag_1_N_2,3,4_ and Ag_1_N_3,4_ configurations, which indicates single N atom doping will induce more stable structures than that of more N atoms doped. The Ag-doped (8,0) ZnO nanotube is distorted compared with the undoped one because the Ag-O bond lengths are longer than the Zn-O bond lengths. For the Ag_1_N_2_, Ag_1_N_3,4_, and Ag_1_N_2,3,4_ configurations, there are bonds between Ag and N atoms. The average bond lengths in these configurations and the bond lengths of Zn atoms and N atoms are displayed in Table 1.

**Table 1:** **Bandgap (*E***_**gap**_**), Zn-N bond lengths (*R***_**Zn-N**_**), and formation energies (*E***_**f**_**) of Ag-N-codoped ZnO nanotubes**

	***E***_**gap**_**(eV)**	***R***_**Ag-N**_**(Å)**	***R***_**Zn-N**_**(Å)**	***R***_**Ag-O**_**(Å)**	***E***_**f**_**(eV)**
(8,0) Ag_1_	1.17	-	-	1.868	0.410
(8,0) Ag_1_N_2_	1.10	1.853	1.838	1.883	0.523
(8,0) Ag_1_N_3,4_	1.20	1.860	1.836	1.893	0.626
(8,0) Ag_1_N_2,3,4_	1.25	1.879	1.833	-	0.719
(8,0) Ag_1_N_5_	1.15	-	1.842	1.870	0.570
(8,0) Ag_1_N_6_	1.17	-	1.846	1.869	0.572

### Electronic properties

**Figure 2:**
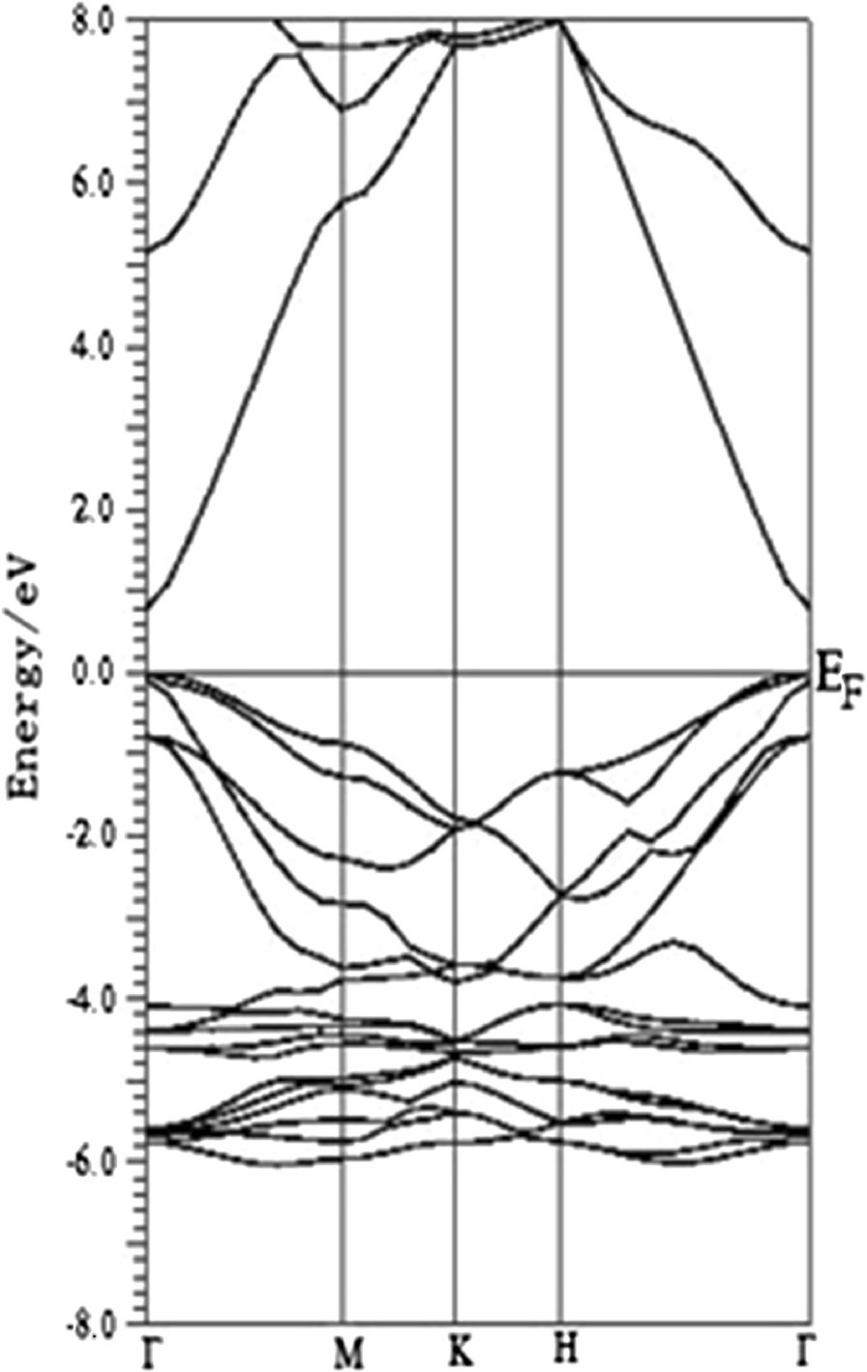
The calculated band structures of 3D bulk ZnO crystal.

**Figure 3:**
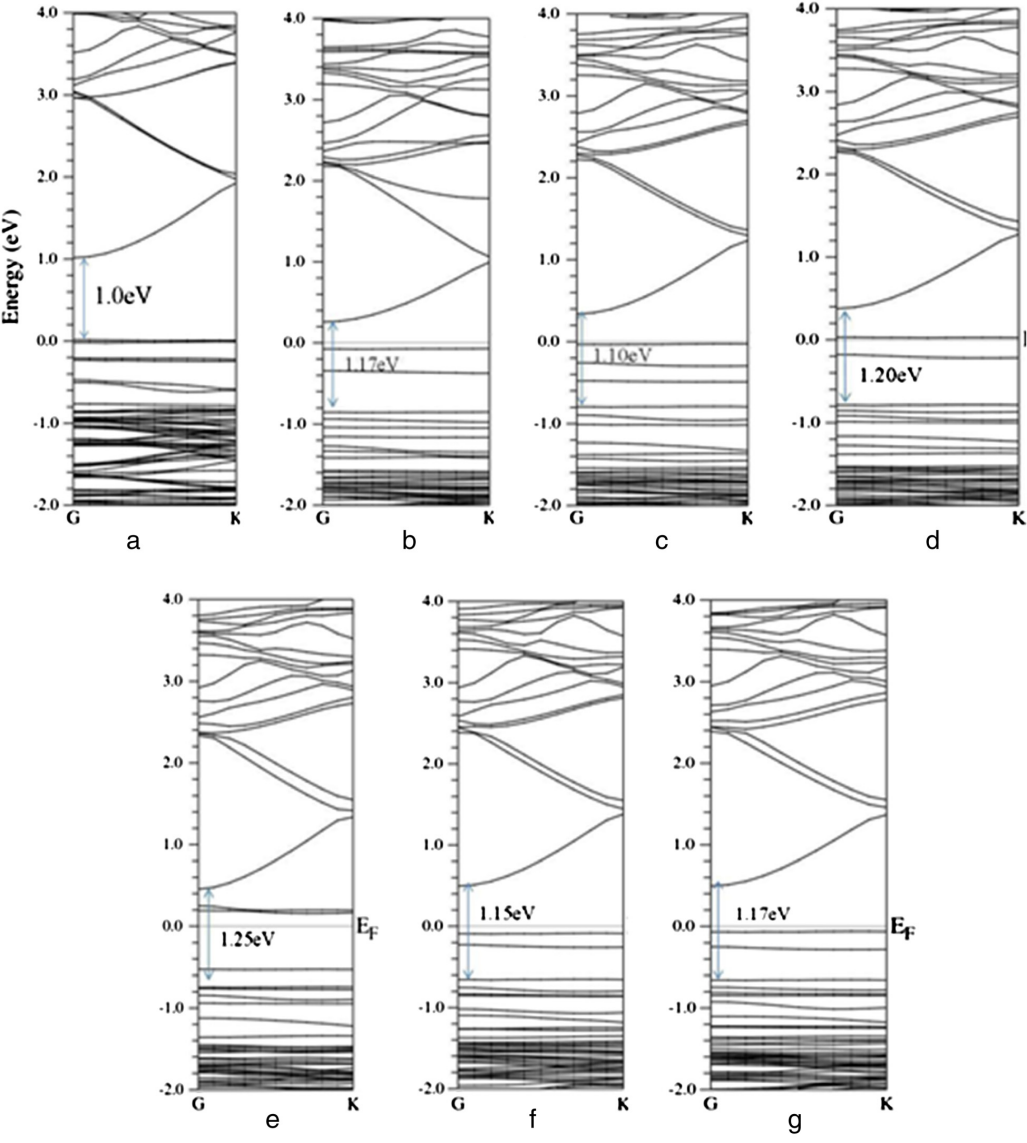
**Band structures of pure and Ag-N-codoped (8,0) ZnO nanotubes. (a)** Pure (8,0) ZnO nanotube, **(b)** Ag_1_ configuration, **(c)** Ag_1_N_2_ configuration, **(d)** Ag_1_N_3,4_ configuration, **(e)** Ag_1_N_2,3,4_ configuration, **(f)** Ag_1_N_5_ configuration, and **(g)** Ag_1_N_6_ configuration.

**Figure 4:**
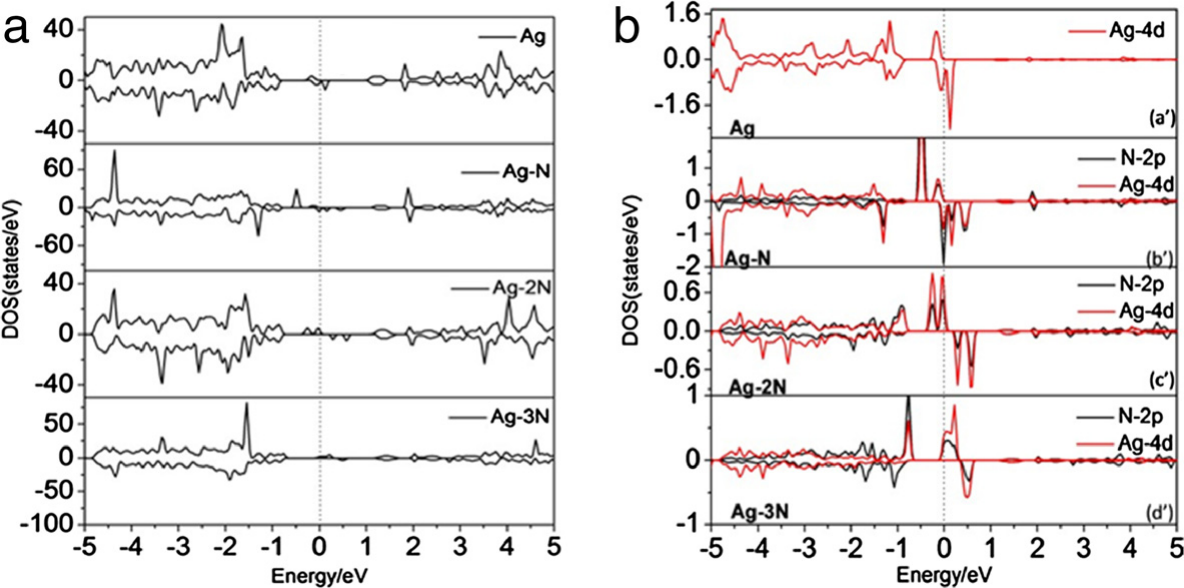
**Total DOS (a) and PDOS (b) of Ag**_**1**_**, Ag**_**1**_**N**_**2**_**, Ag**_**1**_**N**_**3,4**_**, and Ag**_**1**_**N**_**2,3,4 **_**configurations.**

As shown in Figure 2, the further calculation of band structure for bulk wurtzite ZnO shows a direct bandgap of 0.81 eV, which is in good agreement with the previous calculation [18], but is smaller than the experimental value. In Figure 2, the valence band maximum (VBM) of the bulk ZnO is predominantly contributed by O 2*p* character. The conduction band minimum (CBM) basically originates from the Zn 4*s* states with small O 2*p* states. That is to say, the electronic transition from O 2*p* states to Zn 4*s* states is responsible for the optical absorption onset of pure ZnO. For the pure (8,0) ZnO nanotube, the bandgap is 1.0 eV, close to other calculated value of 1.17 eV. The bandgap of ZnO nanotube is larger than the bulk material (0.81 eV) due to the quantum confinement effect. For Ag-doped ZnO nanotube, the bandgap increases to 1.17 eV (shown in Figure 3b), and two impurity levels appear and are located below the Fermi level, which show a donor character. The calculated density of states (DOS) and project density of states (PDOS) in Figure 4a and part (a′) of Figure 4b show that the two impurity levels originate from the Ag 3*d* states. For N-doped ZnO nanotube (configurations Ag_1_N_2_, Ag_1_N_3,4_, and Ag_1_N_2,3,4_), the bandgaps increase with the N concentrations (1.10, 1.20, and 1.25 eV, respectively) increasing. Some levels pass through the Fermi level, indicating that N impurity acts as an acceptor doping in ZnO nanotube. In Ag_1_N_2,3,4_ system, it follows Figure 3e that the host valence band (VB) is surpassed and two gap states are introduced above the VB. The lowest defect level is occupied and locates at about 0.19 eV above the host VBM. Another gap state is occupied and locates at 0.22 eV above the Fermi level. However, the lowest acceptor level in Ag_1_N_3,4_ is occupied and is located at 0.04 eV around the Fermi level. All these results illustrate that Ag_1_N_3,4_ demonstrates the better p-type behavior than the Ag_1_N_2,3,4_ system. For the Ag_1_N_5_ and Ag_1_N_6_ system, the bandgaps are 1.15 and 1.17 eV, which are different to the Ag_1_N_2_ system (1.17 eV), indicating that the bandgap has nothing with the distance of Ag atom and N atom. Before investigating the Ag doping effect on the ZnO nanotubes' optical properties, we calculated the density of states (DOS) of Ag-N-codoped (8,0) ZnO nanotubes as shown in Figure 4, which indicates that Ag-doped ZnO nanotube shows typical characters of p-type semiconductor. Figure 4a,b shows that the states located at the Fermi level are dominated by Ag 4*d* states and N 2*p* states, demonstrating the occurrence of the N 2*p* to Ag 4*d* hybridization. As discussed above, more impurity states will be introduced in the band structure with the increase of N dopant concentration. From Figure 4 (c′), we find that the hybridization between Ag atom dopant and its neighboring host atoms results in the splitting of the energy levels near the Fermi level, which shifts to the majority spin states downward and minority spin states upward to lower the total energy of the system.

### Optical properties

As discussed, the optical properties of pure and Ag-N-codoped (8,0) ZnO nanotubes are based on the dielectric function, absorption coefficient, and reflectivity. In the linear response range, the solid macroscopic optical response function can usually be described by the frequency-dependent dielectric function *ϵ*(*ω*) = *ϵ*_1_(*ω*)+ *iϵ*_2_(*ω*) [19], which is mainly connected with the electronic structures. The real part *ϵ*_1_(*ω*) is derived from the imaginary part *ϵ*_2_(*ω*) by the Kramers-Kronig transformation. All the other optical constants, such as the absorption coefficient, reflectivity, and energy loss spectrum, are derived from *ϵ*_1_(*ω*) and *ϵ*_2_(*ω*).

**Figure 5:**
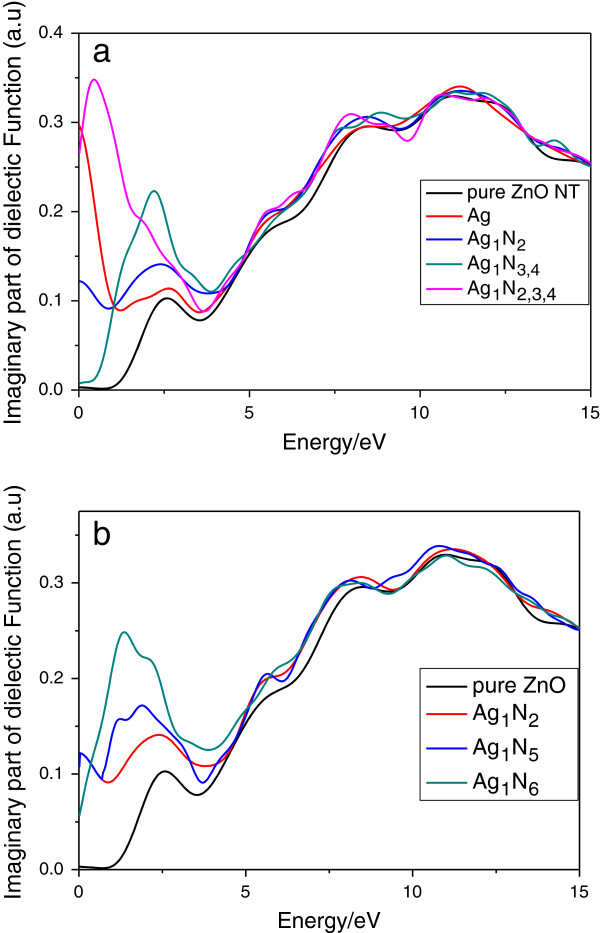
**Dielectric function spectra of pure and Ag-N-codoped (8,0) ZnO nanotubes. (a)** Configurations of Ag_1_, Ag_1_N_2_, Ag_1_N_2,3,4_, and Ag_1_N_3,4_. **(b)** Configurations of Ag_1_N_2_, Ag_1_N_5_, and Ag_1_N_6_.

The calculated dielectric functions of pure and Ag-N-codoped (8,0) ZnO nanotubes are shown in Figure 5. The optical anisotropy are considered in this paper, and we have studied *ϵ*_2_(*ω*) under parallel polarization only, which is named as *ϵ*_2_(*ω*)^p^. In Figure 5a, the pure (8,0) ZnO nanotubes have four peaks located at about 2.6, 8.3, 11.1, and 15.0 eV. The first peak located at 2.6 eV is mainly due to the transition from O 2*p* states to Zn 4*s* states. The second peak at 8.3 eV corresponds to transitions between the Zn 3*d* states and O 2*p* states. The peaks at 11.1 and 15.0 eV are associated with the electron transition between Zn 3*d* states and O 2*s* states. For the Ag_1_ configuration, the peak in the range from 5.0- to 13.0-eV energy region originates from the Zn 3*d* states to O 2*p* states and Zn 3*d* states to O 2*s* states. The peak in the low-energy region at about 0.1 eV mainly comes from the electronic interband transition between Ag 4*d* states and Zn 4*s* states in the conduction band. The peak positions of the Ag_1_N_2_, Ag_1_N_2,3,4_, and Ag_1_N_3,4_ configurations are similar to that of Ag_1_ configuration except that the peaks are more intense because of higher N concentration. The peak at about 2.0 eV originates from the electronic transition from Ag 4*d* states to Zn 4*s* states for Ag_1_ configuration while it originates from the electronic transition from Ag 4*d* to N 2*p* for Ag_1_N_2_, Ag_1_N_2,3,4_, Ag_1_N_3,4_, Ag_1_N_5_, and Ag_1_N_6_ configurations. A red shift occurred for the peak at about 0.5- to 2.0-eV energy region for the Ag_1_N_2_, Ag_1_N_2,3,4_, and Ag_1_N_3,4_ configurations with the increase of N concentration, because the electron transition energy from the occupied impurity states to CBM has a red shift, and the gap of the occupied impurity states to CBM are 0.395, 0.366, and 0.201 eV, respectively. Figure 5b shows the dielectric function spectra of Ag_1_N_2_, Ag_1_N_5_, and Ag_1_N_6_ configurations. In Figure 5b, the peak at 1.0- to 5.0-eV energy regions has a red shift, and the volume of the peak increases with the increasing distance of Ag atom and N atom.

**Figure 6:**
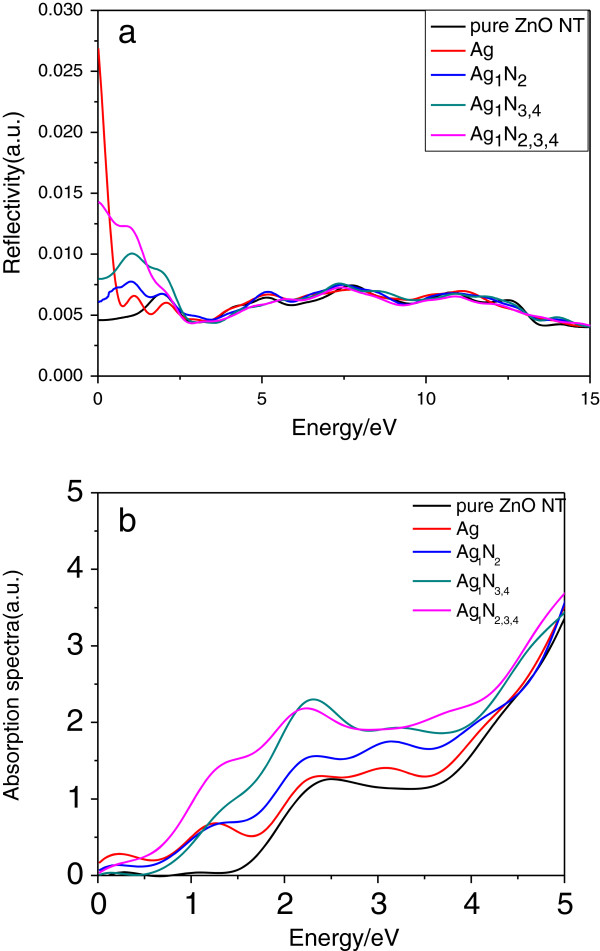
Reflectivity (a) and absorption spectra (b) of pure and Ag-N-codoped (8,0) ZnO nanotubes.

Figure 6 shows the reflectivity and absorption spectra of pure and Ag-N-codoped (8,0) ZnO nanotubes. For the reflectivity of the pure ZnO nanotube, four peaks (located at 2.5, 6.0, 8.0, and 11.6 eV, respectively) can be observed, which correspond to the ones at 2.6, 8.3, 11.1, and 15.0 eV in *ϵ*_2_(*ω*), respectively. For the Ag_1_ configuration, there is a new transition peak near the Fermi energy levels because Ag is doped into the ZnO nanotube, and it is associated with the electron transition between Ag 4*d* states and O 2*s* states. However, the peak at about 2.0 eV (in the visible light region) emerges for the configurations of Ag_1_N_2_, Ag_1_N_2,3,4_, and Ag_1_N_3,4_, which is due to the electronic transition from dopant Ag 4*d* states to N 2*p* states, and it increases with the increase of N concentration. The *R*(*ω*) of the pristine and Ag-N-codoped ZnO nanotube becomes smaller compared to that of the pure ZnO crystal [20]. This indicates that the transmissivity of the ZnO nanotube gets better in the visible light range. The optical absorption calculation shows that the absorption spectra of the Ag-doped and Ag-N-codoped ZnO nanotube become larger than pure ZnO nanotube. The foreign doping atoms in the ZnO nanotube have shifted the absorption edge towards visible light. These results show that doped ZnO nanotube has better optical absorption ability than pure ZnO nanotube in the visible and UV light range.

## Conclusions

In summary, we have studied the structural, electronic, and optical properties of pure and Ag-N-codoped (8,0) ZnO nanotubes using DFT. The configurations with Zn atoms replaced by Ag atoms are p-type semiconductor materials. For the N-doped ZnO nanotube configurations, the bandgap increases with the N concentration. When N atom replaces the second (Ag_1_N_5_) and third neighbor (Ag_1_N_6_) sites for Ag atom, the bandgap has a slight difference with the N that replaced the nearest neighbor site (Ag_1_N_2_). The calculated dielectric function and reflectivity show obvious peaks in the visible light region which are due to the electronic transition from doped Ag 4*d* states to the Zn 4*s* conduction band for the configuration with Ag atoms replacing Zn atoms (Ag_1_) and Ag 4*d* state to N 2*p* state transitions for the Ag-N-codoped configurations, respectively. The peaks at about 0.5- to 2.0-eV energy region for the dielectric function have a red shift with the increase of N concentration. For the reflectivity, the transmissivity of the ZnO nanotube gets better in the visible light range compared with bulk ZnO.

## Competing interests

The authors declare that they have no competing interests.

## Authors' contributions

P-JW and C-WZ conceived the idea and designed the calculated model. X-YF carried out the electronic structure calculations and data analysis. X-JX performed the analysis method of optical properties. All authors read and approved the final manuscript.

## References

[B1] Iijima S Ichihashi T Single-shell carbon nanotubes of 1-nm diameter Nature 1993 8 603 605 10.1038/363603a0

[B2] Balasubramanian C Bellucci S Castrucci P De Crescenzi M Bhoraskar SV Scanning tunneling microscopy observation of coiled aluminum nitride nanotubes Chem Phys Lett 2004 8 188 191 10.1016/j.cplett.2003.11.028

[B3] Zhao M Xia Y Zhang D Mei L Stability and electronic structure of AlN nanotubes Phys Rev B 2003 8 235415

[B4] Lee SM Lee YH Hwang YG Elsner J Porezag D Thomas F Stability and electronic structure of GaN nanotubes from density-functional calculations Phys Rev B 1999 8 7788 7791 10.1103/PhysRevB.60.7788

[B5] Qian ZK Hou SM Zhang JX Li R Shen ZY Zhao XY Xue ZQ Stability and electronic structure of single-walled InN nanotubes Physica E 2005 8 81 85 10.1016/j.physe.2005.07.002

[B6] Shen X Allen PB Muckerman JT Davenport JW Zheng JC Wire versus tube: stability of small one dimensional ZnO nanostructures Nano Lett 2007 8 2267 2271 10.1021/nl070788k 17608442

[B7] Zhou Z Li Y Liu L Chen Y Zhang SB Chen Z Size- and surface-dependent stability, electronic properties, and potential as chemical sensors: computational studies on one-dimensional ZnO nanostructures J Phys Chem C 2008 8 13926 10.1021/jp803273r

[B8] Ozgür U Alivov Ya I Liu C Teke A Reshchikov MA Doan S Avrutin V Cho SJ Morkoc HA A comprehensive review of ZnO materials and devices J. Appl. Phys 2005 8 041301 10.1063/1.1992666

[B9] Kim KK Kim HS Hwang DK Lim JH Park SJ Realization of p-type ZnO thin films via phosphorus doping and thermal activation of the dopant Appl Phys Lett 2003 8 63 65 10.1063/1.1591064

[B10] Ryu YR Zhu S Look DC Wrobel JM Jeong HM White HW Synthesis of p-type ZnO films J Cryst Growth 2000 8 330 334 10.1016/S0022-0248(00)00437-1

[B11] Park CH Zhang SB Wei SH Origin of p-type doping difficulty in ZnO: the impurity perspective Phys Rev B 2002 8 073202

[B12] Wardle MG Goss JP Briddon PR Theory of Li in ZnO: a limitation for Li-based p-type doping Phys Rev B 2005 8 155205

[B13] Yan YF Al-Jassim MM Wei SH Doping of ZnO by group-IB elements Appl Phys Lett 2006 8 181912 10.1063/1.2378404

[B14] Bian JM Li XM Gao XD Yu WD Deposition and electrical properties of N–In codoped p-type ZnO films by ultrasonic spray pyrolysis Appl Phys Lett 2004 8 541 543 10.1063/1.1644331

[B15] Ahn KS Yan YF Shet S Todd D Enhanced photoelectrochemical responses of ZnO films through Ga and N codoping Appl Phys Lett 2007 8 231909 10.1063/1.2822440

[B16] Wu MH Pei Y Zeng XC Planar tetracoordinate carbon strips in edge decorated graphene nanoribbon J Am Chem Soc 2010 8 5554 5555 10.1021/ja1002026 20355698

[B17] Li YL Zhao X Fan WL Structural, electronic, and optical properties of Ag-doped ZnO nanowires: first principles study J Phys Chem C 2011 8 3552 3557 10.1021/jp1098816

[B18] Usuda M Hamada N Kotani T Van Schilfgaared M All-electron GW calculation based on the LAPW method: application to wurtzite ZnO Phys Rev B 2002 8 125101

[B19] Zhang YG Zhang GB Wang YX First-principles study of the electronic structure and optical properties of Ce-doped ZnO J Appl Phys 2011 8 063510 10.1063/1.3561436

[B20] Xie FW Yang P Li P Zhang LQ First-principle study of optical properties of (N, Ga) codoped ZnO Opt Commun 2012 8 2660 2664 10.1016/j.optcom.2012.01.087

